# Considerations about rodent models of binge eating episodes

**DOI:** 10.3389/fpsyg.2014.00372

**Published:** 2014-04-29

**Authors:** Mario Perello, Spring Valdivia, Guadalupe García Romero, Jesica Raingo

**Affiliations:** ^1^Laboratory of Neurophysiology, Multidisciplinary Institute of Cell Biology, Argentine Research Council (CONICET) and Scientific Research Commission, Province of Buenos Aires (CIC-PBA)La Plata, Argentina; ^2^Laboratory of Electrophysiology, Multidisciplinary Institute of Cell Biology, Argentine Research Council (CONICET) and Scientific Research Commission, Province of Buenos Aires (CIC-PBA)La Plata, Argentina

**Keywords:** food intake, homeostatic eating, hedonic eating, event of hyperphagia, mesolimbic system

## Introduction

A binge eating episode is defined as an uncontrolled event of hyperphagia, in which people quickly eat a large amount of food while feeling a sense of loss of control over eating (Wolfe et al., 2009). Binge eating episodes are observed in a variety of human disorders including bulimia nervosa (BN), binge eating disorder (BED), and the binge/purge subtype of anorexia nervosa (AN) (Berger and Tanofsky-Kraff, 2012). Binge eating episodes are also present in overweight and obese people, as well as non-clinical populations under specific circumstances such as stress. The etiology of this behavior is currently unknown. The use of rodent models has been essential for understanding the pathogenesis of many human diseases; however, it is challenging to mimic all features of human binge eating in rodent models (Corwin and Buda-Levin, 2004; Perello et al., 2010a). In particular, these models should not only display the objective characteristics of a binge eating episode, namely the consumption of a large amount of food in a short period of time, but also the subjective characteristics of the feeling of loss of control.

Recently, we examined the neuronal circuitries activated in naïve mice allowed to spontaneously eat a high fat diet (HFD) pellet for 2 h (Valdivia et al., 2014). We found that satiated mice with free access to regular chow rapidly consume a significant amount of HFD when exposed to it, and that HFD intake recruits centers of the mesolimbic pathway, which are known to be activated in human beings displaying binge eating behavior (see below). Experts in the field agreed that our simple model of HFD overconsumption could be relevant for studying neuronal aspects of binge eating behaviors. However, some reviewers argued that it was misleading to describe our model as a model of binge eating. Some criticisms were that our model lacked indications of feelings of loss of control, repeated feeding episodes, escalation of intake over time, a significant level of hyperphagia, and evidence that bingeing occurred in the face of aversive consequences. The notable divergence in the opinion of the journal's reviewers made evident that a comprehensive debate about rodent binge eating models is needed. Here, we briefly present our opinion about the features that a rodent model should fulfill in order to be considered a reasonable model of binge eating episodes and its implications in terms of the neuronal circuits involved.

## Rodent models of binge eating

The features that a model of binge eating episodes should define include the amount of calories eaten and the duration of the event. An empirically based consensus indicates that a 2 h-duration for a binge episode is a reasonable guideline for human studies (Wolfe et al., 2009). This period of time has been extensively used in rodent studies, including ours, since it comprises the entire event of hyperphagia (Berner et al., 2008; Valdivia et al., 2014). In our experience, however, this period of time could be shorter as mice eat ~70% of the total binge intake during the first hour of food exposure. The importance of the amount of calories eaten in the definition of a binge episode has been a very controversial issue for human studies (Wolfe et al., 2009). In contrast, food intake is easily measurable in rodents, and a significant increase in caloric intake is judged as an essential feature in most studies of binge eating in rodents. In our opinion, at least a 2-fold increase of calorie intake, as compared to the control group, appears to be a reasonable criterion to decide whether a test group has experienced hyperphagia (Valdivia et al., 2014). Rapid hyperphagia in a binge eating episode should occur in an uncontrollable manner; however, perceived loss of control is a challenging concept to measure because it is an inherently subjective experience. The emotional state in rodents is an even harder notion to conceptualize, and there is currently no recognized method to assess it. Thus, we consider that the loss of control during an event of hyperphagia cannot be a requirement for a rodent model of binge eating episodes and that this is the most critical limitation in the search of accurate binge eating models.

Given the inability to include a measurable parameter to confirm that hyperphagia is uncontrollable, other features need to be considered in order to define an event of hyperphagia as a binge eating episode in a rodent model. These features include the presence or absence of previous caloric deprivation and/or limited access to palatable diets. In rodents, caloric deprivation promotes compensatory hyperphagia and also entrains the animals to shift their dietary patterns (Corwin et al., 2011). Food deprivation increases the rewarding value of palatable foods, and hyperphagia persists even after animals have reached their energy needs if fasting episodes are sufficiently severe (Perello et al., 2010b; Kim, 2012). Despite the fact that food deprivation-induced hyperphagia in rodents displays some features observed in human binge eating, its use as a model remains controversial because it is considered that binge eating episodes in humans are not usually driven by hunger (Corwin and Buda-Levin, 2004). Indeed, non-clinical populations or BED patients may display binge eating episodes with sufficient or even excess of energy stores; however, AN and BN patients display binge eating episodes under negative energy balance conditions suggesting that some events may involve hunger (Mathes et al., 2009). Also, dieting or food restriction increases the risk of binge eating episodes not only in BN but also in non-clinical populations and BED (Stice et al., 2001). Thus, we think that food deprivation could be included as an experimental manipulation when trying to mimic particular aspects of human binge eating. Secondly, human beings normally prefer bingeing palatable foods (Avena, 2007). *Ad libitum* fed rodents exposed to a palatable food, such as HFD, display a robust event of hyperphagia (Valdivia et al., 2014). Rodents exposed to glucose or sucrose solutions also show binge intake (Avena, 2007). Thus, time-limited access to palatable diets to induce events of hyperphagia in rodents can be useful to study human binge eating. Interestingly, rodent models of carbohydrate- or fat-bingeing show notable behavioral differences during and after the hyperphagia events suggesting that the diet composition is an important factor affecting the implications of the model (Avena et al., 2009).

The frequency of binge eating episodes is a landmark feature of some human eating disorders that can be controlled in rodent models. In particular, repetitive events of hyperphagia can be induced by exposing animals to either repetitive fasting/refeeding events or daily short-term exposure to palatable foods (Corwin et al., 2011). Thus, the use of repetitive events of hyperphagia can be considered when trying to model particular aspects of BN or BED eating behavior in mice. Binge eating episodes are sensitive to stress, and its impact on food intake depends on the nature and duration of the stressor and individual susceptibility (Corwin and Buda-Levin, 2004). Therefore, the use of environmental stressors together with calorie restriction and/or limited access to palatable diets is another important feature that determines the applicability of the model (Corwin and Buda-Levin, 2004). Of note, the above mentioned features are not specified in the definition of a binge eating episode. Thus, we think these features should not be a requirement for models of binge eating in rodents, but relevant to mimic events of hyperphagia found in particular pathologies.

## Neuronal circuits underlying binge eating episodes

Food intake is regulated by an integrated system involving both homeostatic brain circuits that drive food intake depending on energy store levels and hedonic brain circuits that drive consumption based on rewarding properties of foods (Berthoud, 2012). Homeostatic-driven eating occurs under negative energy balance conditions, when circulating factors of energy availability signal to the brain that energy stores are depleted; in contrast, hedonic-driven eating involves cognitive, reward, and emotional factors that induce the consumption of pleasurable foods even when calories are unnecessary (Berthoud, 2012). Importantly, both homeostatic and hedonic brain circuits that drive food intake are sensitive to peripheral factors, including the hormones leptin and ghrelin (Schwartz and Zeltser, 2013).

Neuronal systems controlling homeostatic eating are located mainly in the hypothalamus and brainstem. The hypothalamic arcuate nucleus (ARC) plays an essential role in the regulation of eating as it is highly sensitive to peripheral signal molecules of energy status (Sohn et al., 2013). The ARC contains a set of neurons that express orexigenic factors, including the neuropeptide Y, and another set of neurons that express anorexigenic factors, including pro-opiomelanocortin-derived peptides (Sohn et al., 2013). The ARC neurons likely act as first order neurons sensing peripheral factors and then regulate second order neurons located within the hypothalamus, hindbrain, and the brainstem dorsal vagal complex, which integrates neuronal inputs from the hypothalamus with peripheral hormones and visceral sensory information (Sohn et al., 2013). Thus, homeostatic regulation of food intake involves hypothalamic systems governing intake on a meal-to-meal basis and also brainstem systems regulating meal size and/or frequency. Neuronal circuits controlling homeostatic eating are presumably involved in food deprivation-induced hyperphagia as food deprivation increases and decreases ARC gene expression of orexigenic and anorexigenic neuropeptides, respectively (Schwartz et al., 2000). In contrast, no changes in ARC neuropeptides are observed prior to scheduled-feeding of a palatable food in rodents (Bake et al., 2013).

Hedonic eating involves the dopaminergic pathways emanating from the midbrain ventral tegmental area (VTA), which project to the nucleus accumbens (NAc) in the ventral striatum and other areas such as the amygdala, medial prefrontal cortex, hippocampus, and hypothalamus (Kenny, 2011). The VTA receives projections from many mesolimbic brain nuclei and also taste information from afferent sensory fibers (Kenny, 2011). Acute rewarding stimuli activate dopaminergic VTA neurons, and dopamine release in the NAc potently enhances the drive to obtain palatable foods (Palmiter, 2007). Several studies have implicated the central dopamine system in a variety of human eating disorders (Bello and Hajnal, 2010). In rodents, NAc dopamine signaling increases in response to hyperphagia induced by either food deprivation or limited access to palatable diets (Yoshida et al., 1992; Hajnal and Norgren, 2001). The endogenous opioid system has also been shown to be involved in binge eating in humans, and food deprivation-induced hyperphagia in rodents has been reduced by opioid receptor antagonists (Boggiano et al., 2005; Corwin et al., 2011). In addition, ingestion of palatable foods increases opioid receptor binding within the NAc in rodents (Kelley et al., 2003). In contrast, acetylcholine release from NAc inter-neurons is involved in meal satiation, and a deregulation of this system may be related to sugar-binge eating in rodent models (Avena, 2009).

The homeostatic and hedonic circuits regulating eating are presumably integrated in the lateral hypothalamic area. This area contains orexin neurons highly innervated by hypothalamic and mesolimbic circuits that project widely within the brain (Schwartz and Zeltser, 2013). In rodents, orexin increases food intake depending on the hunger and palatability of the diet (Mahler et al., 2012). Moreover, orexin neurons are activated in response to food deprivation, in anticipation of palatable foods as well as after acute HFD consumption (Mahler et al., 2012). In human binge eating disorders, the role of the orexin system remains unexplored.

## Conclusions

**Figure 1:**
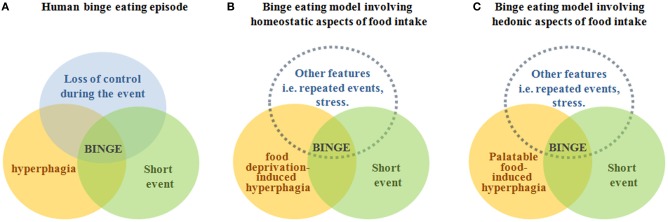
**The figure depicts our point of view of the rodent models of binge eating episodes in relation to the definition of binge eating in humans**. Panel **(A)** displays a diagram representing the definition of an episode of binge eating, which is characterized by a conjunction of hyperphagia of a large amount of food (orange circle), in a discrete period of time (green circle), and with a sense of lack of control over eating (blue circle). Panel **(B)** displays a diagram representing binge eating models in which hyperphagia is induced by food deprivation and presumably involves neuronal circuits controlling homeostatic eating, which are mainly located in the hypothalamus and brainstem (Sohn et al., 2013). Panel **(C)** displays a diagram representing binge eating models in which hyperphagia is induced by the exposure to a palatable food and presumably involves neuronal circuits controlling hedonic eating, which are mainly located in the mesolimbic system (Kenny, 2011). In panels **(B,C)**, circles representing “hiperfagia” and “short event” are drawn with solid lines because they can be clearly defined for rodent models. In contrast, the blue circle representing “other features” is drawn with dotted lines because we think that a “loss of control during the event” circle cannot be required to a rodent model and, instead, other features need to be considered in order to mimic events of hyperphagia found in particular human pathologies.

Clinical reports stress the complexity of assessing if an event of hyperphagia is actually a binge eating episode in humans (Wolfe et al., 2009). Thus, it is not surprising that there is currently not a general consensus in terms of which criteria a rodent model should fulfill to be considered accurate for the study of neurobiological aspects of binge eating episodes. Figure 1 summarizes our opinion in a simple diagram. To conclude, we think that the use of rodent models displaying rapid events of hyperphagia induced by previous caloric deprivation and/or limited access to palatable diets can be useful to investigate the molecular mechanisms and neuronal circuits recruited during binge eating episodes in humans. Importantly, the experimental strategy used to induce the event of hyperphagia and its features determine the main neuronal circuits regulating food intake involved in the model.

### Conflict of interest statement

The authors declare that the research was conducted in the absence of any commercial or financial relationships that could be construed as a potential conflict of interest.
